# Energy Efficiency Optimisation of Joint Computational Task Offloading and Resource Allocation Using Particle Swarm Optimisation Approach in Vehicular Edge Networks

**DOI:** 10.3390/s24103001

**Published:** 2024-05-09

**Authors:** Amjad Alam, Purav Shah, Ramona Trestian, Kamran Ali, Glenford Mapp

**Affiliations:** Faculty of Science and Technology, Middlesex University London, The Burroughs, London NW4 4BT, UK; p.shah@mdx.ac.uk (P.S.); r.trestian@mdx.ac.uk (R.T.); k.ali@mdx.ac.uk (K.A.); g.mapp@mdx.ac.uk (G.M.)

**Keywords:** energy efficiency, meta-heuristic algorithm, vehicular edge computing, particle swarm optimisation, nature-inspired algorithm, task offloading, computation resource allocation, vehicular edge computing

## Abstract

With the progression of smart vehicles, i.e., connected autonomous vehicles (CAVs), and wireless technologies, there has been an increased need for substantial computational operations for tasks such as path planning, scene recognition, and vision-based object detection. Managing these intensive computational applications is concerned with significant energy consumption. Hence, for this article, a low-cost and sustainable solution using computational offloading and efficient resource allocation at edge devices within the Internet of Vehicles (IoV) framework has been utilised. To address the quality of service (QoS) among vehicles, a trade-off between energy consumption and computational time has been taken into consideration while deciding on the offloading process and resource allocation. The offloading process has been assigned at a minimum wireless resource block level to adapt to the beyond 5G (B5G) network. The novel approach of joint optimisation of computational resources and task offloading decisions uses the meta-heuristic particle swarm optimisation (PSO) algorithm and decision analysis (DA) to find the near-optimal solution. Subsequently, a comparison is made with other proposed algorithms, namely CTORA, CODO, and Heuristics, in terms of computational efficiency and latency. The performance analysis reveals that the numerical results outperform existing algorithms, demonstrating an 8% and a 5% increase in energy efficiency.

## 1. Introduction

With the rapid development of wireless technologies and the Internet of Things (IoT) in the field of vehicular networks, there is an increasing need for computational operations to process large amounts of data collected through sensors and other communication systems in connected and autonomous vehicles (CAVs) [1]. These computational operations are required by the vehicles’ onboard systems to manage complex tasks such as route planning, immersive gaming, and vision-based object detection [2]. In addition to the computationally demanding requirements, these applications have significant energy consumption and are delay-sensitive [3,4]. This lowers the overall mileage endurance of the vehicle and affects the quality of service (QoS) as well. However, it is quite tough to fulfil the computational demand of CAVs due to their limited onboard computational power and energy presence.

Offloading the tasks to computational offloading technologies like vehicle cloud computing (VCC), vehicular fog computing (VFC), and vehicular edge computing (VEC) is now an alternative approach to solving the above issues [5]. Although cloud computing infrastructure has been around for a while, resource-intensive applications still have drawbacks, which include costly bandwidth problems, increased latency, and jitter. VFC and VEC computing are two examples that are employed to the edge idea to bring cloud-like resources closer to users. VFC uses adjacent cars as its computing node to determine the best resources [6]. However, a few limitations with fog computing include resource constraints, problems with mobility, and changeable network circumstances [7]. VFC has challenges like coping with optimal performance across a diverse set of vehicles due to the heterogeneity of the vehicles, as it lacks standardisation and interoperability. VFC also has security and privacy concerns due to the exchange of sensitive data between vehicles and fog nodes [8].

Multi-access edge computing in a vehicular environment (i.e., vehicular edge computing) has now been used to achieve the demands of low-latency data transmission and computational activities. This is typically seen in base stations that are positioned close to edge devices [9]. VEC works in a wider range of traffic and mobility scenarios. Hence, the energy optimisation issue can be addressed by using computational offloading technology using VEC computing where tasks are being offloaded for computation toward the edge servers [4,10]. To regulate resource allocation and the trade-offs between energy efficiency and delay management, this article considers the VEC approach. VEC has been taken into consideration as part of a four-layer system framework to enable granular control over VEC functions, including perception, storing, processing, and communication. The minimum assignable resource block (RB) has been taken into consideration to increase the allocation efficiency and resource management performance, as wireless communication plays a major role in the system’s quality of service. In addition, the mobility rate has been considered as a reference variable for this article to address the spectrum’s drift taking place under different moving speeds.

### 1.1. Contributions

The key contributions of this article are listed as follows.

1. A novel four-layer VEC framework is proposed that facilitates more granular control over real-time computation, storage, compatibility, and interconnection of the HetVNets. The use of a four-layer VEC framework will support scalability and would provide better vehicle-related system resources.
2. This article aims to increase overall system energy efficiency by maximising resource allocation and task offloading considering energy constraints.
3. The PSO algorithm has been used for resource allocation and decision analysis (DA) has been used in making optimal decisions towards task offloading. The results has been compared against CTORA, CODA, and Huestristics.

### 1.2. Paper Organisation

The rest of the paper is organised as follows: Section 3 presents the four-layered VEC system framework and the vehicular computation offloading model in VEC (system model). The resource allocation problem formulation and methodology are presented in Section 4. Section 5 illustrates the proposed task offloading and resource allocation scheme. Section 6 presents a comprehensive set of simulation data and interpretation of results, with Section 7 concluding our findings and presenting future directions.

## 2. Related Works

There has been a significant amount of research on task offloading and VEC in recent years within the context of heterogeneous vehicle networks (HetVNets). The aim is to maximise resource utilisation, enhance performance, and achieve energy efficiency. In study [11], two heuristic-distributed and context-aware task offloading approaches (random and exhaustive) have been modelled in order to manage the delay. An online task scheduling method was employed for effective tasks in the edge cloud in [12]. This model was proposed to reduce the communication delay pool. In another study presented in [13], the allocation of computational resources was based on VEC and combined the efficient use of fifth-generation (5G) and short-range communication. In [14], joint optimisation of resource allocation and load balancing was considered in a multi-server multi-user vehicle network. In [15,16], multi-objective optimisation was used to reduce the energy consumption of edge devices and the execution time of computational tasks while preserving privacy. In the work presented in [17], a reinforcement learning-based scheme was implemented on Edge Cloud to find optimal routes for task offloading. In a study from [18], weighted energy consumption was considered in optimising task loading for mobile users. It examined Orthogonal Frequency Division Multiple (OFDM) access and Time Division Multiple (TDM) access with resource allocation on Mobile Edge Computing (MEC). Ref. [19] focused on utilising the mobile edge server by proposing a contract-based computational resource allocation and task offloading. In another study [20], an optimal portion of the workload is considered for offloading, taking factors like workload execution, data transmission, and latency into account. Task offloading and resource allocation are subjects of consideration in many edge computing research studies that address energy efficiency. In [21], optimised resource allocation between the cloud and fog to minimise energy use under different latency constraints. The emphasis on profit optimisation for edge-cloud service providers was presented in [22], where the maximum response time limit and service-level agreements to estimate revenue and penalty costs for each activity were considered. While the above studies focus on low-latency networks and address high-reliability issues such as energy saving, limited onboard vehicle energy has not been thoroughly addressed. In [23], Chang et al. proposed a computational offloading decision optimisation (CODO) to determine the optimal portion of workload to be offloaded based on the dynamic states of energy consumption and latency in workload execution; however, the handover issue was not addressed.

Incorporating cellular and wireless technology, [24] proposed a hybrid VEC in a 5G network for real-time traffic management to maximise the total offloading rate. They addressed a joint power allocation problem, subchannel assignment, and joint task distribution. Ref. [25] presented a low-complexity online algorithm that concurrently determines CPU-cycle frequencies for mobile execution, transmission power for computation offloading, and offloading decisions. The primary objective was to minimise the long-term average execution cost in MEC. The study presented in [26] on downlink spectrum resource management for VEC considered transmission power management among WiFi access points, resource allocation among vehicles, and spectrum slicing in base stations. In some of the above-mentioned studies (e.g., [3,13,14]), the focus was on reducing vehicle energy consumption alongside computational offloading. In other studies (e.g., [12,16,18]), the emphasis was on latency management along with the task offloading issue, without concerns for vehicle energy efficiency. Many studies employed a centralised optimisation method as a prior solution, leading to an issue where the computational complexity dramatically increases with the number of vehicles. This complexity issue can be addressed by adopting a distributed approach to manage energy efficiency and latency. In [27], Fan et al. proposed a joint computational task offloading and resource allocation scheme (CTORA) to minimise the total task processing delay through task scheduling, channel allocation, and computing resource allocation for the vehicles and RSU; however, the work did not make use of 5G.

In [28], a distributed context-aware assignment of tasks is being considered on vehicular networks using a heuristic algorithm to minimise delay. The article [29] combined convolutional neural networks (CNN) with proximal policy optimisation to provide a workload offloading method. They considered tasks lacking strict latency requirements or execution priorities. Ref. [30] examined a task offloading problem involving parked cars acting as servers, using blockchain for decentralised offloading. They proposed and solved this problem using the game system to minimise users’ overall payments. In [31], a Bayesian coalition game to improve energy efficiency and computing resource utilisation in a vehicle cloud was presented. Ref. [32] took into consideration the task offloading issue to reduce the edge server’s communication load. They applied game theory to choose appropriate channels and select the best offloading strategies.

However, some earlier research examined approaches to optimise work offloading or computing resource allocation without optimising both at the same time. For example, the studies reported in [28,29] only looked at task offloading; they neglected to consider computing resource allocation, even though each vehicle was frequently given a variety of computationally demanding real-time tasks. Moreover, task offloading optimisation—which entails unloading the entire work to the MEC server—was ignored in the research in [30,31]. Each vehicle that engages in task offloading chooses whether to unload and where its work will be processed. As a result, vehicles share constrained computing and communication resources. To improve system performance, the job offloading and resource allocation strategies must be optimised. Furthermore, most studies on vehicle task offloading ignore an important component of task offloading and resource allocation with stringent latency limits and energy requirements. We developed a multi-vehicle task offloading game that takes vehicle movement into account in addition to task deadlines and energy consumption constraints, which sets it apart from earlier task offloading techniques for VEC.

Overall, the main research gaps identified are represented below in Table 1.

In this article, we propose using a meta-heuristic Particle Bee Colony Swarm Optimisation (PSO) and decision analysis (DA) algorithm to minimise the overall energy consumption by jointly optimising the computational task offloading and resource allocation algorithm.

We have analysed the computation efficiency problem for CAVs by making an optimised decision on allocating resources and deciding where to upload the tasks. However, designing an efficient offloading approach is tough due to the highly dynamic scenario. Along with the mobility factor, other factors like the required CPU cycle, task data size, and energy consumption will impact the transmission rate and computation efficiency drastically. In this article, computation efficiency has been used as a performance metric which is the ratio of computed bits to the total energy consumed.

We take into considerations the previously mentioned CTORA and CODO for comparison.

## 3. VEC System Framework and System Model

**Figure 1:**
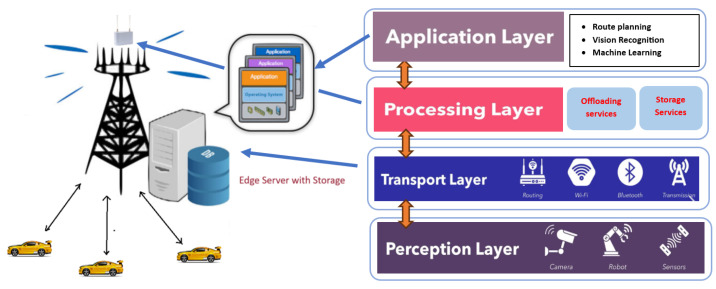
VEC system framework.

Figure 1 below illustrates the 4 layers of the VEC system architecture, i.e., the perception layer, processing layer, transport layer, and application layer.

In Figure 1, the perception layer has been developed to include two types of sensors: an external and an internal system. The internal sensors of CAVs include cameras, millimetre wave radar, Lidar, and other devices, which are the primary focus of current technology. The external sensors, on the other hand, provide extended sensor information from neighbouring vehicles, infrastructure sensors, and the Internet data. The situation awareness capabilities of this layer will aid CAV in planning and decision-making [33]. The communication with all types of vehicles on the road and other RSU units is supported by the transport layer, which is the second layer of our 4-layer approach. The processing layer consists of a storage system, computation offloading service strategy, and decision system. It is primarily responsible for the gathering, processing, and offloading of computations. Intense applications such as intelligent traffic signal management, route planning, and other real-time vehicular onboard Virtual Reality/Augmented Reality (VR/AR), as well as driver behaviour recognition, which can offer immersive services for human–vehicle interactions, are managed by the top application layer.

**Figure 2:**
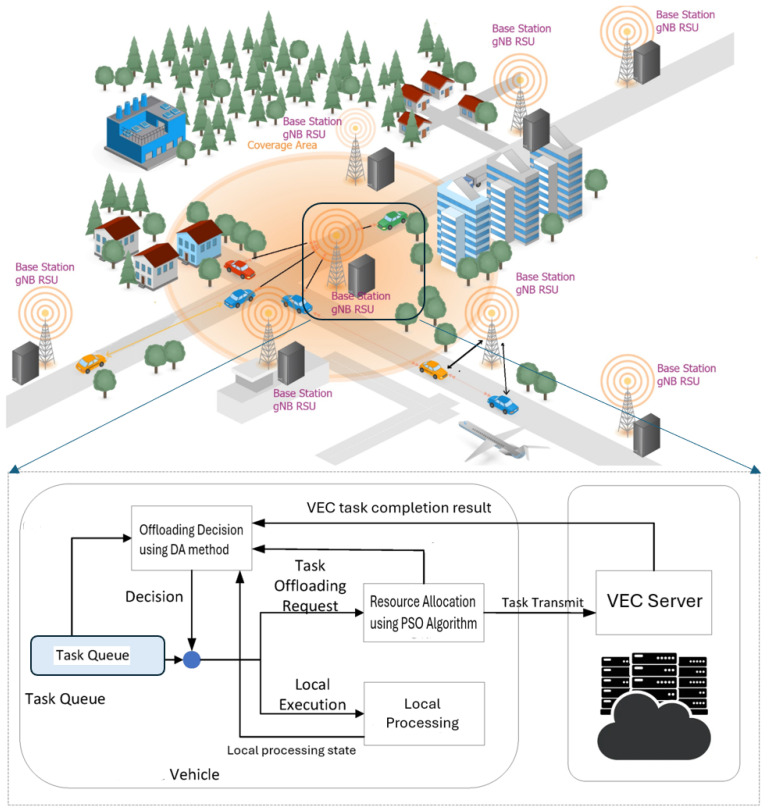
System model.

For vehicle-to-infrastructure (V2I) communication, we consider that each RSU and vehicle can support both cellular (5G-NR-V2X) and millimetre wave-based communication systems. Each RSU and vehicle is equipped with multiple antennas to enable communication over 5G links and mmWave as shown in the system model represented in Figure 2. The communication speed depends on the distance between the RSU and the vehicle. Using mmWave-based V2X within the range of 300 m, a throughput of up to 10 Gb/s can be achieved. Therefore, we have considered a cellular link range to 200 m and mmWave communication range to 150 m [34].

### 3.1. System Topology

In the proposed network we have taken *n* number of vehicles and *m* number of roadside units (RSU’s) in a unidirectional road. The coverage or communication range for all the RSUs has been considered as *r*. Each RSU is integrated with an edge server consisting of $R_{edge}$ computing resources. The vertical distance between the RSU and the road has been considered as *v*. The sets of vehicles have been represented as X=1,2,3,…,n and the set of RSU’s is represented as Y=1,2,3,…,m. Here, we need to consider that each vehicle has some computation tasks that need to be either offloaded to the VEC server or should be computed locally. The offloading decision set is represented here as A=loc,vec. Any vehicle n∈X will connect to RSU m∈Y, provided that the vehicle is in the coverage region of the RSU. In this article, the vehicular offloading strategy is defined as S = $s_{i}$|$s_{i}$ ∈ [ $s_{i}^{loc}$, $s_{i}^{vec}$ ], $s_{i}^{k}$ ∈ 0, 1, : *i* ∈ *X*, *j* ∈ [loc, vec].

### 3.2. Mobility Model

Since the vehicle speed will change dynamically with time along the road, every vehicle has been assigned a random speed vs. chosen from the Gaussian probability density function. Due to the practical nature of traffic, the velocities are bounded away from zero and cannot be negative such as in a congested traffic where vehicles can stop due to traffic signals. Free-flow traffic is considered. As such, a truncated Gaussian probability density function (PDF) applied using the below formula (Equation (1)) with *V* ϵ ($V_{min}$, $V_{max}$) where $V_{min}$ = μ − 3σ and $V_{max}$ = μ + 3σ;

(1) $${\hat{f}}_{V}\left(v\right)=\frac{2f_{V}\left(v\right)}{Err\left(\frac{V_{max}-\mu }{\sigma \sqrt{2}}\right)-Err\left(\frac{V_{min}-\mu }{\sigma \sqrt{2}}\right)}$$

where $f_{V}\left(v\right)$ = [1/$\left(\sigma \sqrt{2\pi }\right)$]$exp(-{\left(v-\mu \right)}^{2}/\left(2\sigma^{2}\right))$ which is the Gaussian PDF. Err() is the error function, σ is defined as a standard deviation of vehicular speed, μ is the average speed, $V_{max}$ is the maximum velocity, $V_{min}$ is the minimum velocity, and *v* is the random chosen velocity of the vehicle [35].

From Equation (1), a corresponding speed ($\mu_{v}$) has been derived, and it lies between ($V_{min}$, $V_{max}$), i.e., $V_{min}$ ≤ $\mu_{v}$ ≤ $V_{max}$ [35].

(2) $$\mu_{v}=\frac{Err\left(\frac{V_{max}-\mu }{\sigma \sqrt{2}}\right)-Err\left(\frac{V_{min}-\mu }{\sigma \sqrt{2}}\right)}{\frac{2}{\sigma \sqrt{2\pi }}\int_{V_{min}}^{V_{max}}\frac{exp(-\frac{{\left(v-\mu \right)}^{2}}{2\sigma^{2}})}{v}dv}$$

where Err() is the error function, σ is defined as a standard deviation of vehicular speed, μ is the average speed, $V_{max}$ is the maximum velocity, and $V_{min}$ is the minimum velocity [35].

The task must be completed before the vehicle leaves the connected RSU and moves to the next RSU on the road. Hence, it is important to know the vehicle’s duration of stay within the connected RSU coverage area. Therefore, the vehicle stay time can be expressed as

(3) $$t_{i}^{hold}=\frac{2\sqrt{r^{2}-p^{2}}}{v_{i}}$$

where *p* is the vertical distance between the road and RSU, the communication radius of its connected RSU is *r*, and $v_{i}$ is the velocity of a vehicle.

### 3.3. Communication Model

mmWave Mode: Each RSU and vehicle is assumed to be installed with directional antenna to have antenna gain. Hence, the transmission rate of vehicle $U_{i}^{\prime }$ can be expressed as

(4) $$U_{i}^{\prime }=B_{mm}log_{2}\left(1+SNR_{i,j}\right)$$

where $SNR_{i,j}$ is the SNR between associated RSU j∈Y in the mmWave mode and the vehicles i∈X and is outlined below in Equation (5) [36].

(5) $$SNR_{i,j}=\left(P_{i,j}-10log_{10}\left(B_{mm}\right)-10\lambda log_{10}\left(\left[r\frac{L_{i}}{r}-L_{i}\right]\right)\right)-SF_{\alpha }-69.6-a_{mm}+A_{i}^{max}A_{j}^{max}$$

where $P_{i,j}$ is vehicle i’s transmission power over its corresponding RSU, $B_{mm}$ is the mmWave channel, λ is the path loss exponent, $\left[r\frac{L_{i}}{r}-L_{i}\right]$ is the distance travelled by vehicle, $L_{i}$ denotes the current position of $vechicle_{i}$, *r* is the communication radius of its connected RSU, $SF_{\alpha }$ is the shadow fading which has been set to 4 dB in line of sight (LOS), $A_{i}^{max}$ is the $vehicle_{i}$ antenna gain, $A_{j}^{max}$ is the $RSU_{j}$ antenna gain, and $a_{mm}$ is the Gaussian noise [37,38].

Cellular Mode: In V2I communication, the cellular link is under NR-V2X. More demanding QoS requirements than those supplied by Cellular V2X can be met by sophisticated V2X applications that NR-V2X can support. Within 5G technology, NR-V2X guarantees enhanced performance in relation to throughput, latency, dependability, connection, and mobility [39].

The data transmission rate between vehicle i∈X and the RSU j∈X m is derived as

(6) $$U_{i}^{\prime }=B_{cc}log_{2}\left(1+\frac{P_{i,j}*d*{\left|\Omega \right|}^{2}}{g_{cc}^{2}}\right)$$

where d is

(7) $$d=(r{\left(\frac{L_{i}}{r}-L_{i}\right)}^{-\theta })$$

$B_{cc}$ is the cellular channel bandwidth, $P_{i,j}$ is the transmission power of the vehicle i∈X, j∈Y, $L_{i}$ represent the vehicle’s current position, $g_{cc}^{2}$ is the Gaussian noise, θ is the path loss, and ${\left|\Omega \right|}^{2}$ is the uplink channel fading coefficient [40].

### 3.4. Computational Model

We can assume that each vehicle will have a computational task along with a maximum acceptable delay, i.e., $T_{i}$ = $C_{i}$, $d_{i}$, $t_{i}^{max}$, where $C_{i}=d_{i}*K_{i}$; $d_{i}$ represents the size of the data block, $K_{i}$ represents the service co-efficient of the vehicle, $C_{i}$ is the computational resource required to complete the task $T_{i}$, and $t_{i}^{max}$ represents the maximum acceptable delay for that task. The threshold acceptable delay should be less than the stay time of the vehicle within the coverage area of the connected RSU. Hence, the acceptable delay will be min $\left[t_{i}^{max},t_{i}^{hold}\right]$.

1. Local computational time and energy consumption: If the vehicles need to compute the task locally, then the time taken, and the energy consumed to complete the task are presented below.

(8) $$t_{i}^{local}=\frac{C_{i}}{\alpha_{i}^{local}}$$

and

(9) $$e_{i}^{local}=C_{i}*\tau_{i}$$

where $\alpha_{i}^{local}$ is the vehicle own computing resource, and $\tau_{i}$ represents the energy consumed per task.

2. Time and energy consumption on the VEC: The vehicles need to offload the task to VEC, if the local computation is not feasible. Here, we need to add the uplink transmission time along with VEC execution time. Hence the time taken and the energy consumed to complete the task on VEC are presented below.

(10) $$t_{i}^{VEC}=\frac{C_{i}}{\alpha_{i}^{VEC}}+\frac{d_{i}}{U_{i}}$$

where $\alpha_{i}^{VEC}$ is the computational resource assigned to any connected vehicle by VEC, and $U_{i}$ is the vehicle upload data transmission rate.

The total energy consumed in the system ($E_{i}$), i.e., energy consumed by the vehicle i ∈ X, while offloading the task to VEC and energy consumed on VEC while executing the assigned task by the vehicle i.

(11) $$E_{i}=e_{i}^{local}+p_{i}*\frac{d_{i}}{U_{i}}+\Delta E_{loss}$$

where $P_{i}$ represents the average transmission power of $vehicle_{i}$, and $\Delta E_{loss}$ is the energy lost due to the multipath fading effect.

3. Energy time tradeoff (ETT): In optimisation or decision-making situations, ETT is defined as the weighted sum of energy consumption and task execution time to reflect the tradeoff between the utilisation of energy resources and the time to accomplish a specific objective, i.e., the vehicle’s task computation requirement. Hence, the ETT for any vehicle i for local computation can be represented as

(12) $$ETT_{i}^{local}=\beta *e_{i}^{local}+\gamma *t_{i}^{local};\;ETT\;for\;local\;computing$$

(13) $$ETT_{i}^{VEC}=\beta *e_{i}^{VEC}+\gamma *t_{i}^{VEC};\;ETT\;for\;VEC\;computing$$

where β and γ indicate weights of task executing time and energy consumption for vehicle i such that 0 ≤β≤ 1 and 0 ≤γ≤ 1.

In the above ETT for local and VEC computing, the values of β and γ can be selected by the vehicle according to the priority needed on energy or time. For example, vehicles can select a higher β value, if they have a higher energy priority or can select a higher γ value if they have a higher time priority.

In addition to ETT for local and VEC computing, we also need to find max($ETT_{i}^{local}$) which is the task’s maximum energy consumption and tolerable delay, if executed locally. We also need to find max($ETT_{i}^{VEC}$) which is the task’s maximum energy consumption and tolerable delay on VEC, if executed on VEC.

(14) $$max\left(ETT_{i}^{local}\right)=\beta *e_{i}^{max(local)}+\gamma *t_{i}^{max(local)};$$

(15) $$max\left(ETT_{i}^{VEC}\right)=\beta *e_{i}^{max(VEC)}+\gamma *t_{i}^{md};$$

where $t_{i}^{md}$ is the maximum manageable or acceptable delay

4. Offloading decision-making function: The vehicle needs to decide whether the tasks should use its local computational resources or to offload to VEC. For making this decision, the below functions are used to guarantee the execution within the maximum acceptable delay.

(1) Decision Function for local execution: The local computing decision function $DF_{i}^{local}$ for a vehicle i can be represented as

(16) $$D_{i}^{Local}=ln(1+max\left(max\left(ETT_{i}^{local}\right)-ETT_{i}^{local}\right)-\psi b(max\left(ETT_{i}^{local}\right)$$

provided that $max\left(ETT_{i}^{local}\right)<ETT_{i}^{local}$, where ψ is used to keep the condition satisfied that $ETF_{i}^{local}$ should be less than $s_{i}^{VEC}*ETT_{i}^{VEC}$. The function b(.) is used as a boolean function that will either return 0 if $max\left(ETF_{i}^{local}\right)<ETT_{i}^{local}$ is false, or it is equal to 1, if it is true.

(2) Decision Function for VEC execution: The VEC computing decision function for a vehicle *i* can be represented as $DF_{i}^{VEC}$ for a vehicle *i*

(17) $$D_{i}^{VEC}=\mu_{i}*ln\left(1+max\left(max\left(ETT_{i}^{VEC}\right)-ETT_{i}^{VEC}\right)\right)-\left(1-\mu_{i}\right)*\zeta_{vec}*\alpha_{i}^{VEC}$$

where $\zeta_{vec}$ is the unit per computing cost on VEC, $\mu_{i}$ is the weight coefficient of the decision function, and $\alpha_{i}^{VEC}$ is the resource allocation on VEC for a vehicle [41].

## 4. Problem Formulation and Methodology

In this section, we need to frame the total computational efficiency which is defined as the ratio of the total computed bits to the vehicle’s energy usage. The total computation efficiency (TE) of the whole system can be framed as

(18) $$TE=\sum_{i=1}^{X}\sum_{j=1}^{Y}\frac{DF_{i}^{j}*S_{i}^{j}*T_{i}}{E_{i}}$$

We now need to address energy efficiency and computational enhancement of the whole system. The goal of optimisation is to minimise the total energy consumption of the system by maximising the total efficiency function (TE) in Equation (16). In order to achieve this, we create an optimisation problem that maximises the system’s utility by optimising resource allocation and the task offloading technique which can be expressed as

(19) max(TE)

provided that the following requirements are met such that

(20) $$s_{i}^{VEC}\left(max\left(ETT_{i}^{VEC}\right)\right)+s_{i}^{local}\left(max\left(ETT_{i}^{local}\right)\right)\geq s_{i}^{j}*ETT_{i}^{j}$$

R1: $\sum_{i=1}^{X}\alpha_{i}^{vec}\leq R_{i}^{VEC},\;\forall_{i}\in X$
R2: $s_{i}^{local}+s_{i}^{VEC}\leq 0$ where R = {$\alpha_{1}^{vec}$, $\alpha_{2}^{vec}$, $\alpha_{3}^{vec}$, $\alpha_{4}^{vec}$......$\alpha_{X}^{vec}$} is all the resource allocation on VEC, and S = {$s_{1}$, $s_{2}$, $s_{3}$...$s_{n}$} is the execution vehicle offloading strategy.

As the above Functions (17) and (18) involves sum-of-ratio maximisation, the Particle Swarm Optimisation (PSO) algorithm [42] has been used for computation resource allocation. The decision analysis (DA) algorithm has been used for the vehicles’ offloading decisions. Once the offloading decisions take place, then the optimisation of computation resource allocation on VEC takes place using the Particle Swarm Optimisation (PSO) algorithm.

After the resource allocation stage, the DA algorithm modifies the task offloading mechanisms until an optimal point is reached. To maximise the utility of each offloading vehicle, the computation resource allocation of VEC computing must be optimised once all vehicles have made their offloading decisions. The system reaches the nearly ideal solution through mutual iteration and reaches a steady state.

### Particle Swarm Optimisation (PSO) Algorithm

Particle Swarm Optimisation (PSO) is a computational approach used in computer science to optimise a problem by repeatedly attempting to improve a candidate solution in relation to a specified quality metric. To obtain an improved solution, the particle swarm optimisation technique uses a cluster of particles. By using a population of potential solutions, referred to as particles in this instance, and manipulating their position and velocity inside the search space, it solves the problem. Using basic formulas, these particles are shifted around in the search space. Both the best-known position of the entire swarm and the particles themselves serve as guides for their travels in the search space. These will eventually start to direct the swarm’s motions as better sites are found. A workable solution is eventually found if the procedure is repeated [42]. Additionally, in this method, N particles are initialised by the population, and each particle has a unique position a, velocity $v_{a}$, and personal best position $P_{{best}_{i}}$.

As stated above, every particle updates its position and velocity, particularly by learning from $g_{{best}_{i}}$ and $P_{{best}_{i}}$, the global and personal best positions. The new positions can be represented by the following equation.

(21) $$\left\{\begin{array}{c} v_{a^{new}}=\delta *\left(v_{a^{old}},x_{a^{old}},p_{{best}_{i}},g_{{best}_{i}}\right)\\ x_{a^{new}}=x_{a^{old}}+v_{a^{new}} \end{array}\right.$$

From the above Equation, $x_{a^{new}}$ and $v_{a}^{new}$ indicate the position and new velocity of the ath particle in the present iteration where δ represents the velocity updating approach in PSO. In the next equation, $x_{a}^{old}$ and $v_{a}^{old}$ are considered as the position and velocity of the old ath particle in the preceding iterations. It was noticed that if $x_{a}^{new}$ was better than the previous old position, then the new best position is replaced by $x_{a}^{new}$. Consequently, for particles that find a better position, their counts are reset, whereas for the particles that fail to update $p_{{best}_{i}}$, their count will be significantly increased.

In the next step for performing a better search, the particles with better fitness values are selected. Further, the fitness value for every particle is computed on the basis of the best position with the following equation.

(22) $$fit\left(x_{a}\right)=\left\{\begin{array}{c} \frac{1}{1+f(p_{{best}_{i}})},if\;f\left(p_{{best}_{i}}\right)\geq 0\\ fit\left(x_{a}\right)=1+\left|f\left(p_{{best}_{i}}\right)\right|,otherwise \end{array}\right.$$

The probability $p_{a}$ for the selection of the ath particle is calculated as the following equation.

(23) $$P_{a}=fit\left(x_{a}\right)/\sum_{a=1}^{n}fit\left(x_{a}\right)$$

The particles were selected on the basis of probability pa by utilising the roulette method. Further, the particles which have better $p_{{best}_{i}}$ can possibly be selected. When assuming that the sth particle $x_{a}$ was selected, Equation (26) will be utilised for generating the new position $x_{s}^{new}$. If this new position was better than ${p_{b}est}_{s}$, then the ${p_{b}est}_{s}$ will be replaced by $x_{s}^{new}$. Subsequently, and for particles that fail to update their ${p_{b}est}_{s}$, their counter will be significantly increased.

The particles that fail to update its $p_{{best}_{i}}$ in some iterations are considered exhausted and are abandoned. Velocity and position as well as $p_{{best}_{i}}$ are randomly initialised in the search space.

So the position and velocity updating equations in PSO algorithm can be represented with the following equation.

(24) $$\left\{\begin{array}{c} v_{a}^{new}=w.v_{i}^{old}+c.rand.\left(pbest_{\tau a}-x_{a}^{old}\right)\\ x_{a}^{new}=x_{a}^{old}+v_{a}^{new} \end{array}\right.$$

In the above equation, rand indicates the random vector within [0, 1], w indicates the inertia weight, and c is said to be the learning factor. τa and $p_{{best}_{\tau a}}$ indicate the index vector for the ath particle.

## 5. Our Proposed Task Offloading and Resource Allocation Scheme

### 5.1. Task Offloading

The decision of the vehicle to offload a task depends on its offloading demands and offloading strategies of other vehicles. In this paper, the use of the decision analysis (DA) algorithm will help to make the decision feasible and ensure convergence, thereby making an optimal decision.

The DA method is used to formulate the multivehicle computing problem. In this problem, every vehicle is considered as a player and has needs for computational tasks. Players in this game will create a decision tree to maximise the payoff (i.e., resource utility). For vehicle *i*, the decision strategy function is given as *D*($s_{i}$, $s_{-i}$), where $s_{-i}$ = ($s_{1}$, $s_{2}$..., $s_{i-1}$, $s_{i+1}$,..., $s_{n}$) denotes the other vehicles’ offloading strategies except vehicle i. The objective of each vehicle is to choose a valuable offloading strategy.

The task offloading strategy DA is described as

(25) $$\max_{s_{i}}D\left(s_{i},s_{-i}\right)=s_{i}^{vec}*D_{i}^{vec}+s_{i}^{loc}*D_{i}^{loc}$$

where vehicle set is *n*, and the set of offloading strategies of vehicle *i* is denoted by $D_{i}$.

Therefore, all vehicles calculate the expected payoff and look for the maximum payoff. Through mutual iteration, the system enters into a steady state and achieves the near-optimal solution.

### 5.2. Computation Resource Allocation

This section aims to maximise the vehicles’ utility by offloading the tasks to the VEC server and the optimum resource allocation can be derived by the using the below formula.

(26) $$\max_{R}\;\Sigma \;\mu_{i}*ln\left(1+max\left(max\left(ETT_{i}^{VEC}\right)-ETT_{i}^{VEC}\right)\right)-\left(1-\mu_{i}\right)*\zeta_{vec}*\alpha_{i}^{VEC}$$

For solving the above resource allocation problem, we have used the particle swarm optimisation (PSO) algorithm which is a metaheuristic optimisation algorithm [42] in which a population of particles (i.e., vehicles), each of which represents a possible solution to an optimisation problem which direct the search process towards the best solution.

The process of the PSO optimisation has been presented in Algorithm 1 below.
**Algorithm 1: Resource allocation and offloading strategy****Decision Strategy Calculation using DA method**1. To start with n number of vehicles, i.e., *X* = {1, 2, 3, ..., *N*}2. for all vehicle *i* ϵ *X* repeat            3. calculate the offloading strategy using Equation (15) (Solving using DA method)            4. if $\left(D^{VEC}>D^{local}\right)\;D^{VEC}=1\;and\;D^{local}=0$            5. if $\left(D^{VEC}<D^{local}\right)\;then\;D^{VEC}=0\;and\;D^{local}=1$            6. calculate the total energy of the system, i.e., TE from Equation (16)7. end of for**Resource Allocation Calculation using PSO method**8. Starting with again n number of vehicles, i.e., *X* = {1, 2, 3, ..., *N*} andProblem Formulation: Considering other factors as stated in Section 3.4: $T_{i}$ = $C_{i}$, $d_{i}$, $t_{i}^{max}$, where $C_{i}=d_{i}*K_{i}$; $d_{i}$ represents the size of the data block, $K_{i}$ represents the service co-efficient of the vehicle, $C_{i}$ is the computational resource required to complete the task $T_{i}$, and $t_{i}^{max}$ represents the maximum acceptable delay for that task.9. Particle Representation: Randomly choose a swarm of particles, for each particle, initialise position and velocity (particle’s position represent parameters such as vehicle speed).10. Define the fitness function, often denoted as f($x_{i}$), *i* = 1, ……, *N*; where *x* represents a particle’s position in the search space (Here fitness function related to the objective of the optimisation problem).11. Let $pbest_{i}$ be current best position, let also $g_{best}$ be current best position among all particles.12. while the convergence condition is not satisfied do13.    for each index *i* = 1→*N* do14.        Determine the global best position $gbest_{a}$ among all particles15.        Update the particle velocity $v_{a}$ and the position $x_{a}$ using $v_{i}\left(t+1\right)$ = ω* $v_{i}\left(t\right)$ + $c_{1}$ *$r_{1}$*($pbest_{i}$ − $x_{i}\left(t\right)$) + $c_{2}$ *$r_{2}$*(gbest − $x_{i}\left(t\right)$)16.        Update new position f($x_{i}^{t+1}$) = $x_{i}^{t}$ + $v_{i}\left(t+1\right)$17.        Evaluate the fitness of each particle’s new position using the fitness function: If $x_{i}^{new}$ is better than $pbest_{i}$ then step 10 otherwise step 1118.           $pbest_{i}$ = $x_{i}^{t+1}$19.        Evaluate the fitness of each particle’s new position using the fitness function: If $x_{i}^{new}$ is better than $pbest_{i}$ then step 12 otherwise step 1320.           $gbest_{i}$ = $x_{i}^{t+1}$21.        endIf22.      endfor23.           Return the best solution found, represented by the global best position $gbest_{i}$ for obtaining resource allocation as specified in Equation (18).

$r_{1}$ and $r_{2}$ are random numbers. $c_{1}$ and $c_{2}$ are cognitive and social parameters, respectively. ω is the inertia weight. $v_{i}\left(t\right)$ is the velocity of particle i at time t. $g_{best}$ is the global best particle among all particles, and $pbest_{i}$ is the personal best position of particle i. Finally, $x_{i}\left(t\right)$ is referred as the current position of particle I [42].

## 6. Results

In our system model, RSUs broadcast beacon messages to all the vehicles on computation resource information in their communication range. All the vehicles also periodically share their relevant information with the RSUs, and once the connection is established, communication goes into the unicast mode between the RSUs and vehicles. In this simulation, we have ignored communication overhead, as the size of the message is too small in comparison to the higher bandwidth used in 5G NR-V2X.

### Performance Analysis

Using MATLAB software, 2022b the performance of the proposed algorithm has been evaluated in comparison with different algorithms, i.e., the computation task offloading and resource allocation (CTORA) algorithm, the computation offloading decision optimisation (CODO) algorithm, and the heuristic scheme algorithm.

Our proposed work is evaluated against the CTORA, CODO, and heuristic scene algorithm.

The heuristic scheme [43] allows work to be offloaded to the VEC server when a vehicle’s time and energy restrictions cannot be met by doing computation locally. In this algorithm, other cars are not taken into consideration throughout this process. In the CODO scheme [44], the tasks are either performed locally or offloaded to the VEC server using the computation offloading decision optimisation scheme. The primary distinction between our proposed method and CODO is that our method took mmWave communication into account. Finally, the CTORA method [45] solely focuses on optimising the decisions related to offloading inside a specific computing resource.

In this section, we present the numerical findings of our proposed algorithm. In this scenario, vehicles are simulated to move in a single direction. We have considered six RSUs in a line, and each RSU has a VEC server along with it. In the simulation, we have used [15, 20] GHz as the computation resource for each vehicle.

The detailed settings of other simulation parameters are summarised in Table 2.

**Figure 3:**
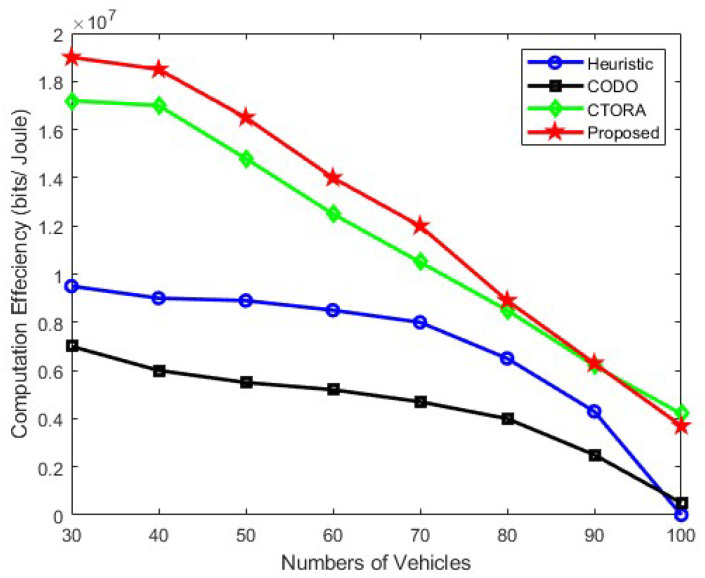
Computation efficiency vs. varying number of vehicles for communication performance.

Figure 3 shows the computational efficiency with respect to the number of vehicles and illustrates the impact of communication performance with the varying number of vehicles on computational efficiency. For these results, the data size of the task has been kept at 1 kilobit, and the vehicle speed has been maintained at 45 km per hour. The locations of the vehicles around the RSU have been considered random. Upon analysis of the results, it can be observed that as the number of vehicles increases, computational efficiency decreases for all algorithms as well as the proposed algorithms. The decrease in computational efficiency is attributed to various factors such as the location of the vehicles, SNR, and task data size. Depending on the vehicle’s location, the signal-to-noise ratio decreases with the increased vehicle distance from the RSU. Computational efficiency also depends on the communication technology, such as the use of mm waves and cellular waves. From the results, it can be seen that the CODO Scheme performs worse due to its lack of utilisation of mm wave communication. Additionally, it is observed that the heuristic scheme performs better than the CODO scheme and provides better performance until the vehicle count exceeds 95. Finally, it is also evident that our proposed scheme performs the best of all, even when the number of vehicles is up to 95, but there is slight fall in the performance after 95.

**Figure 4:**
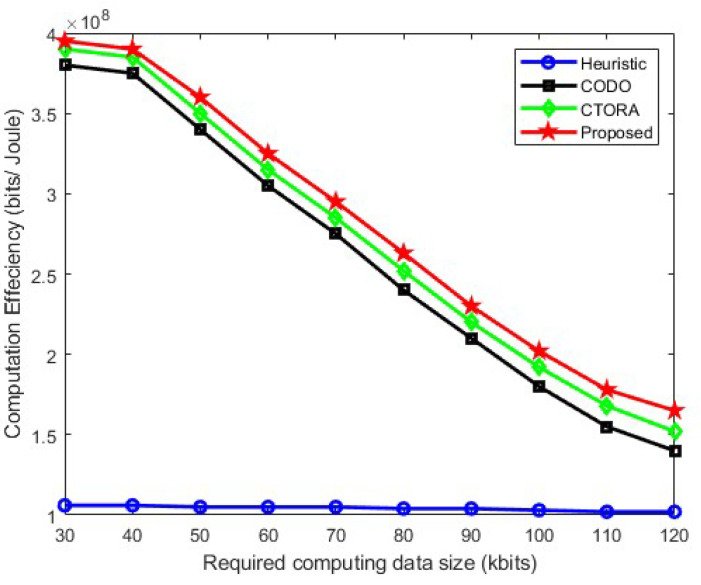
Computation efficiency vs. computing data size needed.

In Figure 4, the computational efficiency is analyzed with respect to the required computational data size used during communication. It can be observed from the results that our proposed scheme performs better than the others, i.e., CODO and CTORA. Furthermore, it is evident that as the required computing data size increases, the computational efficiency decreases due to various reasons. Firstly, processing times and latency increase as a result of handling higher data quantities, demanding more processing power and memory. Secondly, higher data volumes may potentially cause network congestion, requiring more bandwidth to send and receive data packets, leading to packet loss and re-transmissions. Additionally, the need for more sophisticated data compression, routing, and management methods due to larger data sizes further increases computational cost. In Figure 4, it can be seen that the computational efficiency for our proposed scheme is closer to the others when the required computing data size is smaller. However, as the computation data size increases, our proposed scheme exhibits a decrease in computational efficiency; although, it remains superior to others. In the heuristic approach, offloading decisions are made by vehicles if energy constraints and local computation time fail to meet requirements, resulting in lower and relatively unchanged computational efficiency. The local computation is deemed the best option when the required computing data size is smaller, as offloading depends on available bandwidth and channel gain between the RSU and vehicles. Conversely, offloading to the VEC server becomes the preferred option when the required computing data size increases.

**Figure 5:**
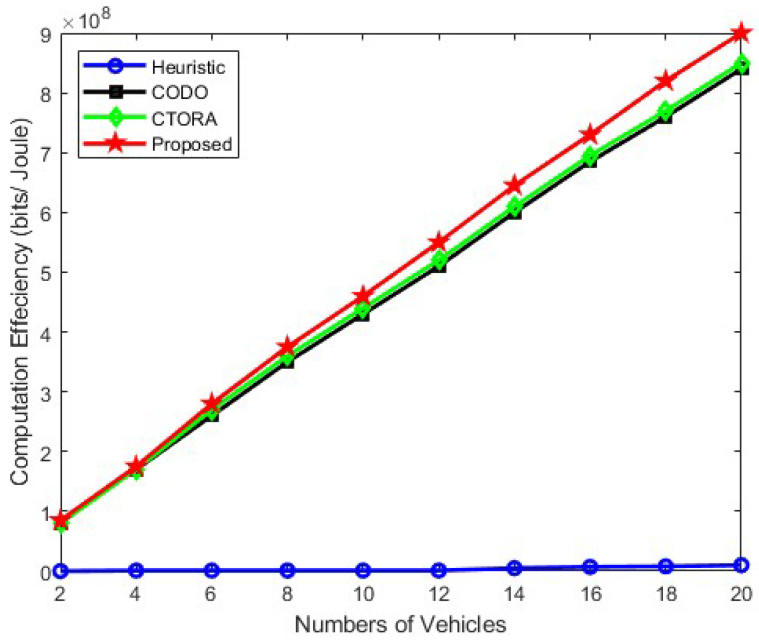
Computation efficiency vs. varying the number of vehicles under speed of 25 km/h.

**Figure 6:**
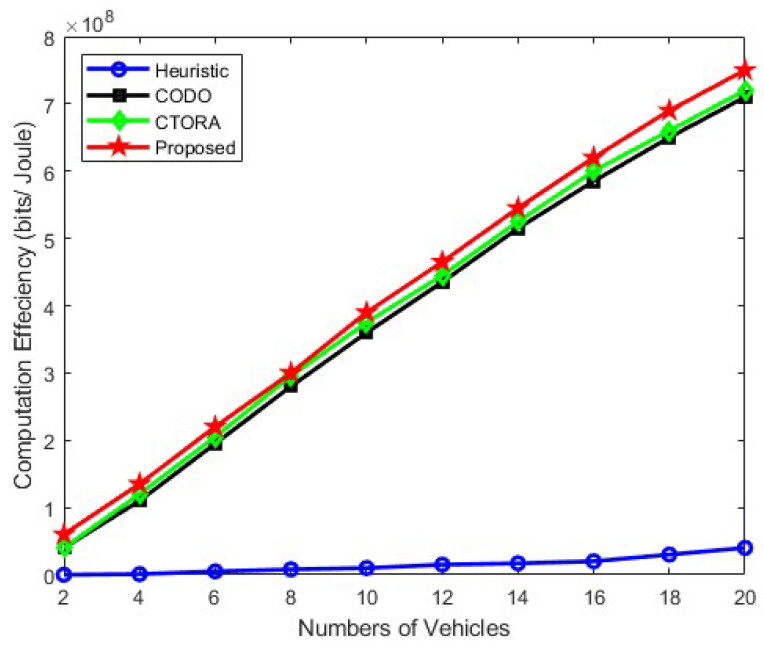
Computation efficiency vs. varying the number of vehicles under under speed of 45 km/h.

**Figure 7:**
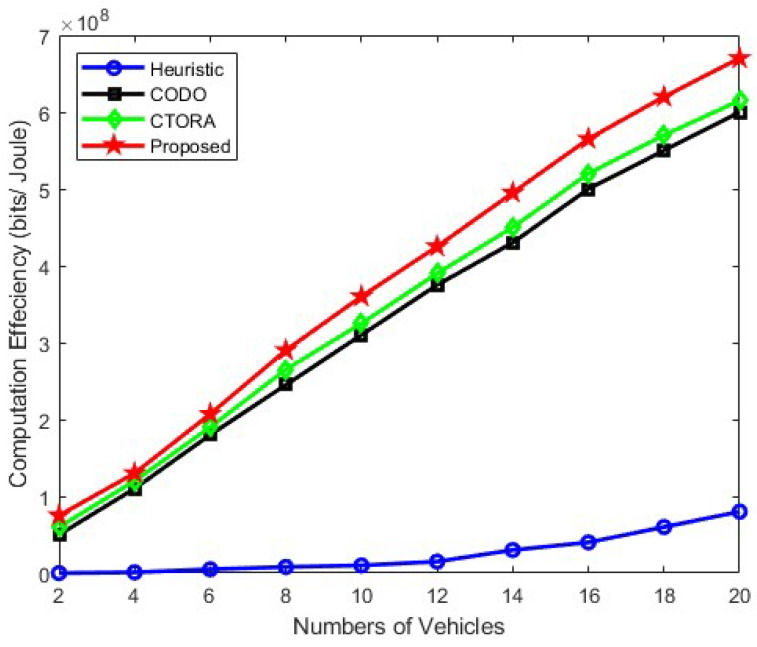
Computation efficiency vs. varying the number of vehicles under under speed of 65 km/h.

Figure 5, Figure 6 and Figure 7 illustrate the computational efficiency when the number of vehicles ranges from approximately 2 to 20, maintaining an average speed of 25 km/h, 45 km/h, and 65 km/h, respectively. In this analysis, the required computational data size remains constant and uniform across all vehicles. Notably, the heuristic schemes operate at a diminished capacity, as vehicles tend to prioritise local computation to minimise practical tolerable delay in Figure 5. Conversely, other schemes, namely CODO and CTORA, perform commendably, alongside our proposed scheme. In Figure 6 and Figure 7, the efficacy of our proposed scheme surpasses that of CODO and CTORA, attributed to the integration of mmWave communication and optimised resource allocation strategies, enhancing the overall performance and efficiency and shows a stable gain. A slight gain has been observed in Figure 6 and Figure 7 for Heuristic method, but the gain is too low in comparison to other models. Similar trend is been observed in Figure 6 and Figure 7, for CODO, CTORA, and our proposed model where an average speed of 45 km/h and 65 km/h has been taken into consideration. Notably, our proposed model does shows similar gain. Our suggested scheme also works well in conjunction with CODO and CTORA, the other two schemes. Hence, it shows that the use of the PSO algorithm does help in optimised resource allocation and thereby improving the overall performance and efficiency.

**Figure 8:**
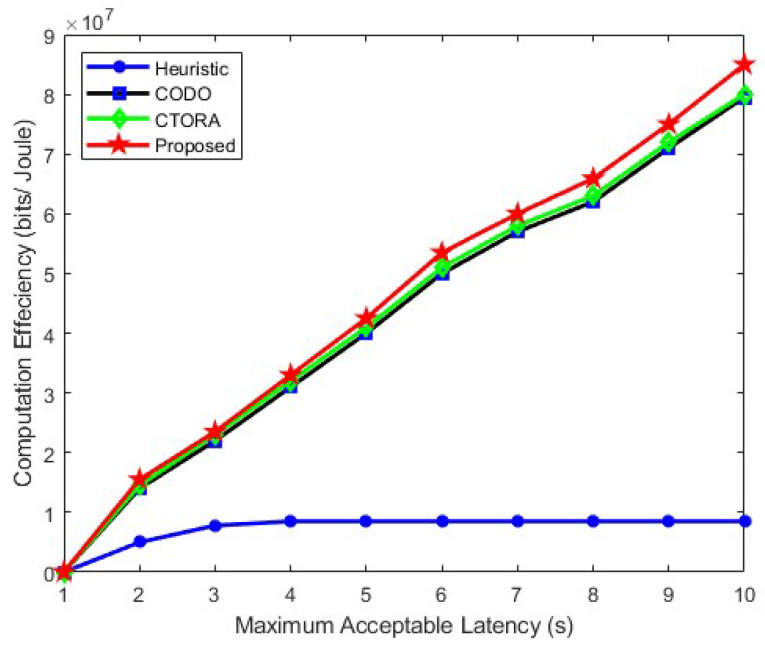
Computational efficiency vs. maximum acceptable latency.

Figure 8 presents an analysis of computational efficiency with regard to the maximum tolerable delay. The findings indicate a notable trend: as the maximum tolerable delay increases, there is a corresponding decrease in the total energy consumption. In this context, both the CODO, CTORA, and our proposed algorithm exhibit a strong performance compared to heuristic approaches. This can be attributed to their shared strategy of prioritising the offloading of most tasks to the VEC server when the maximum tolerable delay is set at 2. Conversely, the heuristic algorithm tends to favour local computation once the maximum tolerable delay surpasses 2. A distinguishing feature of our proposed algorithm is its superior performance when compared to other algorithms (heuristic, CODO, and CTORA) as the maximum tolerable delay increases further. Despite this advantage, the performance gap of our proposed algorithm aligns closely with that of the CODO and CTORA algorithms; although, the disparities widen slightly by 8% and 5%, respectively. This suggests that our proposed algorithm maintains competitiveness and performed well, particularly in scenarios with higher maximum tolerable delays.

## 7. Conclusions

Within the context of a tradeoff between computing time and energy consumption, we examined a vehicle’s strategy to offload duties in order to maximise computation efficiency in this research. We combined job offloading and computation resource allocation to construct the computation efficiency problem. Our PSO approach combined with the DA method allowed us to accomplish an efficient resource allocation and task offloading. In addition, we made use of mmWave and cellular link in the 5G NR-V2X communication paradigm to enhance system communication latency and performance. According to the numerical results, the suggested technique shows an increase in computation efficiency adhering to energy and computation time limits. Hence, the comparison of different algorithms with respect to the proposed algorithm, i.e., the PSO optimisation algorithm, is shown in Figure 4, Figure 5, Figure 6 and Figure 7. Among them, the average energy consumption of the PSO algorithm shows an enhancement in all. In comparison, we can see that the proposed algorithm (PSO algorithm) consumed less energy in comparison to CTORA, CODO, and heuristic; however, CTORA and CODO seems to be the same or very close at the start, but on the further end of each result, our proposed model showed an enhancement in comparison to other algorithms. In general, the suggested method, PSO optimisation with the DA method demonstrates superior performance compared to other algorithms, while keeping 1KB packet sizes. We would expand our research in the future to investigate and use other optimisation approaches such as Sequential Quadratic Programming (SQP) or the Genetic Algorithm approach to enhance the long-term delay performance and fortify the job offloading procedure along with the combined use of mmWave and 5G NR communications.

## Figures and Tables

**Table 1:** Research gaps.

Works Cited	Contribution	Gaps
[12,16,18]	Emphasis was on latency management along with the task offloading issue	No concern for vehicle energy efficiency
[24]	Proposed a hybrid VEC in a 5G network to maximise the total offloading rate	No concern for joint optimisation of resource allocation
[25,26]	Efficient task offloading employed and resource allocation	No joint optimisation on task offloading and resource allocation
[27,28]	Optimisation of task offloading and resource allocation	Optimisation was focused only on MEC server

**Table 2:** Table below represents all the simulation parameters used in this article.

Parameter Used	Value
Cellular link Bandwidth	25 MHz
mmWave Bandwidth	250 MHz
Number of RSUs’	6
RSU coverage length	250 m
Communication range for mmWave and cellular links	150 and 250 m
Input data size	[30, 80] kB
Maximum latency constraint	[0.1, 1] s
Vehicles arrival rates	0.1 vehicles per second
Vehicle (Vi) transmission rate ($\left(p_{i}\right)$)	1.5 W
Average velocity of Vehicles	45 km/h
Computational resource price/VEC	0.05 4/GHz
Vehicle Antenna gain ($A_{i}$)	20 db
RSU antenna gain ($A_{j}$)	20 db
Energy consumption/computing unit	2 × 10^−10^ w
Input data size	[30, 60] kB
Path loss exponent (λ)	3.1

## Data Availability

The data presented in this study are available on request from the corresponding author.
